# The relationships between relative age effect, personality constructs and achievement level in soccer

**DOI:** 10.3389/fspor.2023.1226599

**Published:** 2023-11-10

**Authors:** Sofie Bolckmans, Kris Perquy, Janet L. Starkes, Daniel Memmert, Werner F. Helsen

**Affiliations:** ^1^ Institute of Exercise Training and Sport Informatics, German Sport University Cologne, Cologne, Germany; ^2^ Sport Consultancy MP Mental Coaching in Topsport, Sport and Performance Psychologist RBFA, Tubize, Belgium; ^3^ Department of Kinesiology, McMaster University, Hamilton, ON, Canada; ^4^ Department of Movement Sciences, KU Leuven, Leuven, Belgium

**Keywords:** individual differences, long-term career, well-being, underdog hypothesis, talent identification

## Abstract

**Introduction:**

Youth soccer academies are challenged with the constant recruitment process of young talented players to select those who will achieve long-term success as an athlete. Youth soccer academies strive to enhance the physical and technical skill development as well as personality development of talented players because psychological characteristics play a crucial role in players’ future success in their transition to professional soccer. The least mature players and relatively young players may have a greater need to possess superior technical/tactical or psycho-behavioral skills than those relatively older counterparts because of the higher selection rates of early maturing players. Due to RAEs, a significant decrease in the overall quality of professional soccer teams might be observed because of the loss of talent of physically smaller, but psychologically stronger and more versatile relatively young players who possess proper technical and tactical attributes at an early age. The first objective of this study was to examine any possible relationship between RAE and personality constructs. A second objective was to identify factors and effects that can help in the further improvement of talent selection and equal opportunities for elite youth soccer players based on their underlying RAE. The third objective was to consider the impact of RAE on long-term career development.

**Methods:**

In this retrospective observational study, 151 elite youth soccer players between 15 and 18 years of age were first grouped in birth quartiles. Personality constructs were then assessed, using a combination of observations, interviews, and a self-assessment questionnaire. Next competition level after 8 years was evaluated to identify RAEs, differences in personality characteristics and opportunities to reach professional soccer player status between relatively older vs. younger players.

**Results:**

A clear significant RAE was observed for the whole database (Q1 = 38.4% vs. Q4 = 13.9%) with OR of 2.61 (*χ*^2^ = 19.46, *p* < 0.01, *r* = −0.85). Relatively young players had higher median scores on personality constructs such as self-confidence (*p* = 0.04), while relatively old players had higher median scores on personality constructs such as team orientation (*p* = 0.03). In the long term, more players of the youngest birth quartile were signed as professional players (76.2%), compared with relatively old players (46.6%). 65.0% of the 20 players had the highest total score on personality constructs developed as a professional soccer player, vs. 35.0% of the 20 players with the lowest scores.

**Discussion:**

In conclusion, this study showed not only further evidence of the RAE but also provided evidence supporting “the underdog hypothesis” in national elite youth teams. Relatively young players were also more likely to get higher value senior professional contracts in the long term. We propose that this may be due to the relatively young players developing superior psychological skills and technical expertise to compensate for their early physical disadvantage. This in turn suggests the need for greater awareness of the importance of personality constructs in the future development of youth elite soccer players. Therefore, the crux of the issue is how youth soccer academies elicit the “best of both worlds” ie. moderating RAE whilst also gaining the benefits of the underdog hypothesis by creating the right environment for every player to develop to their full potential in elite youth soccer academies.

## Introduction

Youth soccer academies, are constantly challenged with recruitment of young talented players to select those who will achieve long-term success as an athlete (1–4). Because of their continuous growth process, children are most often grouped according to their chronological age in education and sports settings, including soccer (5). In the Flemish educational system, pupils are also organised into one-year age groups, using January 1 as the cut-off date. Although, maturational effects during puberty may be responsible for potentially large development differences between chronological and biological age, described as relative age effect (RAE) (5–7). This is especially true for sports where physical characteristics are important, as in soccer, which then results in a selective advantage for those with early maturation (6, 8, 9).

Despite the crucial role of physical performance in talent selection, other areas of performance (technical, tactical, psychological, or cultural) are as important to fully assess a player's quality and long-term development potential. A multifactorial assessment is is essential (1–3, 10).

The first aim of this study was to examine a possible relationship between RAE and personality characteristics. A second purpose was to identify factors that can help improve talent selection and equal opportunities for elite youth soccer players given their underpinning RAE. Next, the impact of RAE on long-term career development for this group of players was assessed, with the hypothesis that relatively younger players that are selected to play on an elite team have the advantage of receiving higher quality soccer education, described as the “underdog hypothesis” (11).

The RAE in sports is a global phenomenon and is present in the majority of sports where physical characteristics are important (11–13). Selection biases because of the RAE, likely lead to homogenous pools of players selected for academy soccer programs who are often either relatively old and/or who are early maturing in comparison to population norms (6, 9, 14, 15). These early maturing players are frequently characterized as possessing temporary, maturity-related advantages both in anthropometric (e.g., stature and body mass) and physical fitness characteristics (e.g., power, strength, speed) (7, 16–18).

Coaches and scouts have the intention to select relatively old players who are physically stronger or bigger at the time of selection and are therefore more likely to be perceived as “talented” and subsequently selected for talent development programmes without considering their long-term potential (1, 2, 16, 18, 19). This compromises the selection of players with greater potential in the long-term, who were born towards the end of the year (19). Helsen et al. (19) also demonstrated that relatively old children were more likely to get extra opportunities such as advanced levels of coaching, transfers to better teams, and more frequently being selected for experiential opportunities, or becoming a professional soccer player.

Boccia et al. (20) revealed that most players in elite senior teams were not selected for elite youth teams before, which suggests that junior-to-senior transition is not determined by youth national team selections. The RAE influences strongly the selection for national youth teams, but its impact was clearly smaller in the youth-to-senior transition. Brustio et al. (21) revealed that only a few players, selected in the Italian female national youth teams, reached the Italian female national senior team. They also remarked that the RAE in U17 and U19 with a playing position's effect in the younger age categories, became smaller in the national senior teams.

Conversely relatively young children were more likely to drop out early (9, 19). In the long-term, a clear decrease in the overall quality of the highest competitive teams will be present since talented players with proper technical attributes are overlooked at an early age due to a lack of physical development which is simply related to the period of the selection year in which they were born. Selection in talent development systems is not only associated with receiving better education, experience, and coaching, but also facing stronger opponents. Because higher competition is more prestigious and challenging, it is also likely to increase one's motivation and self-esteem (1, 6, 19, 22, 23).

A combination of physical, cognitive, emotional, and motivational factors accumulate to produce RAEs and maturity selection bias (1, 4, 6, 24). Many promising and talented players have been overlooked in the past because they suffered from a relative age disadvantage in their early childhood often during selection procedures in talent academies (19). The long-term effect of RAEs likely produce a significant decrease in the overall quality of the highest competitive professional teams since smaller, psychologically stronger players with proper technical attributes are overlooked at an early age due to a lack of physical development, that is simply related to the period of the selection year in which they were born.

It should be recognized that the determinants of the RAE are multifactorial (14, 25, 26). Psychological characteristics, that underlie the RAE in greater depth through various social agents, have been integrated into models of talent identification and development as significant predictors of success in sports of players (12).

The way in which these social agents (parents, coaches, and athletes) interpret mechanisms such as physical stature, maturity, and cognitive ability creates RAEs through various effects such as the Pygmalion, Galatea, and Matthew effects (12). As such it is essential that the influence on psychological and cognitive parameters also needs to be factored in, since we know that psychological factors are important to scouts and coaches during the talent selection process (1, 2). The development of physical performance skills stays a crucial element of talent identification. However, other parts of performance (technical, tactical, psychological, or social) are also important selection parameters to properly evaluate a player's quality and potential long-term development and can assist talent identification and development (1, 4).

Youth soccer academies are the main talent development institutions for professional youth soccer all over the world. Their main aim is to recruit young talented players with the potential to be developed into professional soccer players and to achieve long-term success. Therefore, it is important to better understand why certain players are more likely to be selected into an academy, and also why others might be more likely to successfully graduate as a long-term successful player. The current study provides further evidence of the relative age effect within national elite football teams but goes further and demonstrates an association between the RAE and personality constructs in elite Belgian youth soccer players. It is important to identify personality characteristics for long-term success as an early- or late-maturing soccer player so that the most talented youth soccer players receive continued progressive support from a young age to achieve their maximum potential (22, 27–29). The relationship between the RAE and the long-term career development of the elite youth soccer players was determined. Current literature suggests that the RAE reverses throughout the career of athletes, with relatively younger players having more opportunities to reach professional status (30).

The current state of empirical research shows that psychological characteristics and skills such as motivational orientation, self-reflection, self-regulation, self-confidence and competition anxiety differ between youth players of different performance levels (22, 31, 32). Kavussanu et al. demonstrated that differences between players were largely due to higher performing players’ greater task orientation (33). Previous studies in soccer determined self-confidence was relevant for high performance (32, 34). Concerning emotional stability, previous research has focused on anxiety as an important factor that can influence higher soccer performance (32). Also self-regulation has been found relevant for future performance (22). Höner et al. revealed significant relationships between psychological components such as motivation, volition and self-referential cognition with future performance level; whereas competition anxiety revealed only a weak relationship with performance level (35, 36). Previous research has also demonstrated that elite youth soccer players possess more adaptive self-regulation than non-elite players, suggesting that self-regulation contributes significantly towards success in sport (22, 37).These authors also reported that elite players showed higher levels of reflection and effort, and appeared more willing to invest effort into task execution and adapting their knowledge and actions in order to execute skills (22). Likewise, lack of self-regulated skills has been shown to negatively impact performance outcomes in sport (38).

This study provides further evidence that psychological skills training is essential to both improving and increasing the consistency of performance; which in turn is of benefit to the development of the player, coach, and team homogeneity (39). The findings also support the importance of specific psychological skills training within different levels of maturity, development, and position in the soccer team (1, 27).

The complex nature of the talent development process, together with the multifactorial characteristics associated with superior talent development and the successful transition from youth academy level to senior professional player status, suggests that personality and psychological characteristics are very important in soccer because they may influence all the athlete's performance subcomponents and opportunities to develop as a professional player.

## Methods

### Study population

A retrospective observational study was conducted on 154 male elite Belgian youth soccer players (aged between 15 and 18 years), born between 1990 and 1996 (1990: *n* = 1, 1991: *n* = 19, 1992: *n* = 27, 1993: *n* = 32, 1994: *n* = 32, 1995: *n* = 22, 1996: *n* = 18), who were playing in the Belgian national youth teams U16-U19. Ethical approval was obtained (008185).

Players’ charts were reviewed and data were collected regarding date of birth. Data were collected between March 2010 and February 2012. Three players were excluded from data analysis (deceased (2 players; traffic accident, sudden cardiac death), incomplete data (1 player)). These deselections resulted in a sample of 151 elite Belgian youth soccer players.

At the time of data collection, all players were involved in the Belgian national youth teams. The data were originally collected by the Royal Belgian Football Association (RBFA) to improve coaches’ individualized approach to players and to help players enhance their mental capabilities.

### Study design

Players were grouped within each category according to the Belgian domestic soccer season birthdate quartiles (Q1: January 1st to March 31st; Q2: April 1st to June 30th; Q3: July 1st to September 30th; Q4: October 1st to December 31st) and represented as a percentage of the sample population. Players’ date of birth was collected from charts of the Royal Belgian Football Association (RBFA) and categorized into birthdate quartiles (Q).

### Design of the psychological assessments

During the first phase, the youth soccer players were interviewed and analyzed by two sports psychologists between March 2010 and February 2012 according to guidelines stating that psychological characteristics should be evaluated by combining players’ self-rating and external expert rating in talent development and psychological analysis (27). The players also performed a self-assessment by filling out the “Mental Potential Questionnaire” and the “Task & Ego Orientation in Sports Questionnaire” (TEOSQ) (40). These questionnaires are based on the NEO-FFI-3 Questionnaire, which contains 60 questions and provides an accurate measure of the 5 domains of personality (neuroticism, extraversion, openness, agreeableness, conscientiousness). It can help you to understand a player's emotional, interpersonal, attitudinal, experiential and motivational approach. The players can complete the questionnaires by indicating how much they agree with each statement by entering an appropriate score (1: strongly disagree, 2: disagree, 3: neutral, 4: agree, 5: strongly agree). It is a dimensional representation of personality structure to analyze personality disorder scales. The “Mental Potential Questionnaire” is based on “The Athletic Coping Skills Inventory” (“ACSI”) and is a highly validated psychology assessment that measures an athlete's psychological coping skills in training and competition in seven key areas coping with adversity, coachability, concentration, confidence and achievement motivation, goal setting and mental preparation, peaking under pressure and freedom from worry. Players will answer a list of 28 questions surrounding the 7 different personality constructs, by which personality constructs are measured by 4 questions each. These questions are answered by means of a 4-points scale (0: almost never, 1: sometimes, 2: often, 3: almost always). The final score for each subscale can range from 0 to 12, while the final summation of all the scores for each skill creates a value ranging from 0 to 84 called the Personal Coping Resource. Higher scores in these tests are indicative of the ability to cope with the demands of the sport and to possess greater psychological skills and personality constructs. Afterward, each player was observed by the two sports psychologists during two matches and three training sessions. The psychologists observed the youth players independently from one another before comparing notes and agreeing on a consensus score on the different personality characteristics of each player. The personality constructs of the players were analyzed by two experienced sports psychologists, which are trained to make an objective evaluation of the different personality constructs without being influenced by external factors like the opponent or match outcome. Moreover, players are observed during two matches and three training sessions in order to evaluate how players manage their emotions and concentration in different match and training situations, against different opponents and by different match outcomes. Finally, a one-hour competency-based interview was conducted with each player using the critical incident technique (CIT) (41). In that interview, the soccer players were questioned about six personality characteristics: self-confidence, winning mindset, self-development, managing emotions, concentration, and team orientation. By giving examples of situations in which those constructs were applied during training sessions and matches, the psychologist assessed the personality constructs of the players. The CIT is based on the recall of an actual event in order to examine the likely behavior of players in certain situations. The technique is useful when it is likely that attitudes or behavior would be less likely to be revealed using a direct approach. During a final discussion, the psychologists combined data from the observations, questionnaires, and interviews to score the youth soccer players on the six personality constructs, with each method accounting for one-third of the total score. For each personality construct, players were scored on a nine-point standard scale. A final score ranging between 1 and 3, will indicate that the player's mental skill is insufficiently developed and will have a negative impact on their performance as football player. A score between 4 and 6, will indicate that this mental skill is sufficiently developed, comparable to most elite players and contributes to their performance in a positive way.

Finally, a score between 7 and 9, can indicate that this personality construct is a real strength, in which you are better than most other players.

Based on the player's results, they will receive an individual report with evaluations and recommendations developed by our licensed sport psychologists.

During the second phase, in February 2019, the players’ current playing level was evaluated. They were divided into the following competition levels: professional players abroad, first division Jupiler Pro League, second division 1B, amateur, lower rankings, or no club. This allowed us to examine the potential correlation between their personality constructs, their birthdate quartile, and the highest level of competition they reached in their long-term career development.

### Personality constructs

Psychological constructs, which include personality traits and psychological skills, are relevant predictors of future soccer performance in talent development (1, 27).

The six characteristics were chosen by two experts in the field of sports psychology and the RBFA. They reflect the personality characteristics which play a crucial role in players’ future success. Personality traits are defined as a predisposition to behave in a certain way. Definitions are based on descriptions from the ACSI and “The Big Five” (42, 43).

The six constructs were defined as:

- Self-confidence: showing faith in one's skills, the courage to meet difficult situations, and the pleasure one has in playing soccer
- Winning mindset: the ability to make efforts and to demonstrate discipline to achieve challenging goals, the will to win, the motivation to succeed, and the dedication to the sport. It also includes perseverance after a setback.
- Self-development: showing insight into one's strengths and pitfalls, accepting advice and feedback from others, willing and daring to question oneself, and taking responsibility for one's development.
- Managing emotions: positively using one's emotions and performing under pressure.
- Concentration: the ability to focus on a task and not be distracted by external or internal factors.
- Team orientation: integrating into the group and making a positive contribution to the team atmosphere. It also involves clear and constructive communication, giving advice and feedback to teammates, motivating others in case of setbacks, and showing that group interests predominate over individual interests.

### Statistical analysis

The 151 players were grouped within categories according to typical Belgian domestic soccer season birthdate quartiles depending on their date of birth and expressed as a percentage of the sample population with a cut-off date of January 1st. Statistical analyses were performed to examine the relative age effect of the whole data set. Results were represented as median values. RAE was determined using odds ratios (OR). To verify if there was an RAE, the results were compared with the birth rates of the general Belgian population from 1990 to 1996. The Chi-square test, the Kolmogorov-Smirnov test, and the correlation coefficient were applied to ensure the significance of the RAE results (*p* < 0.01).

Subsequently, the median of the scores for each personality construct was calculated for every birth quarter as well as for the first and last half of the year (semester). The one-tailed Mann–Whitney *U* test was conducted to check if there was a significant difference (*p* < 0.05) between the corresponding medians.

A contrastive analysis was performed on the players’ long-term playing level: the 20 best and 20 worst scoring players on the personality characteristics were identified and compared, concerning the current competition level. The same analysis was also performed on the current playing levels per birthdate quarter, to evaluate which birthdate quarter players achieve more frequently senior professional contracts.

A multiple stepwise regression analysis was carried out to investigate which parameters contribute independently the most to predict professional future performance.

## Results

151 elite soccer players between 15 and 18 years old were grouped within each category according to the Belgian domestic soccer season birthdate quartiles between 2010 and 2012 with a re-evaluation of the long-term playing level after a mean follow-up time of 8 years.

### Do these data also show a relative age effect?

**Figure 1:**
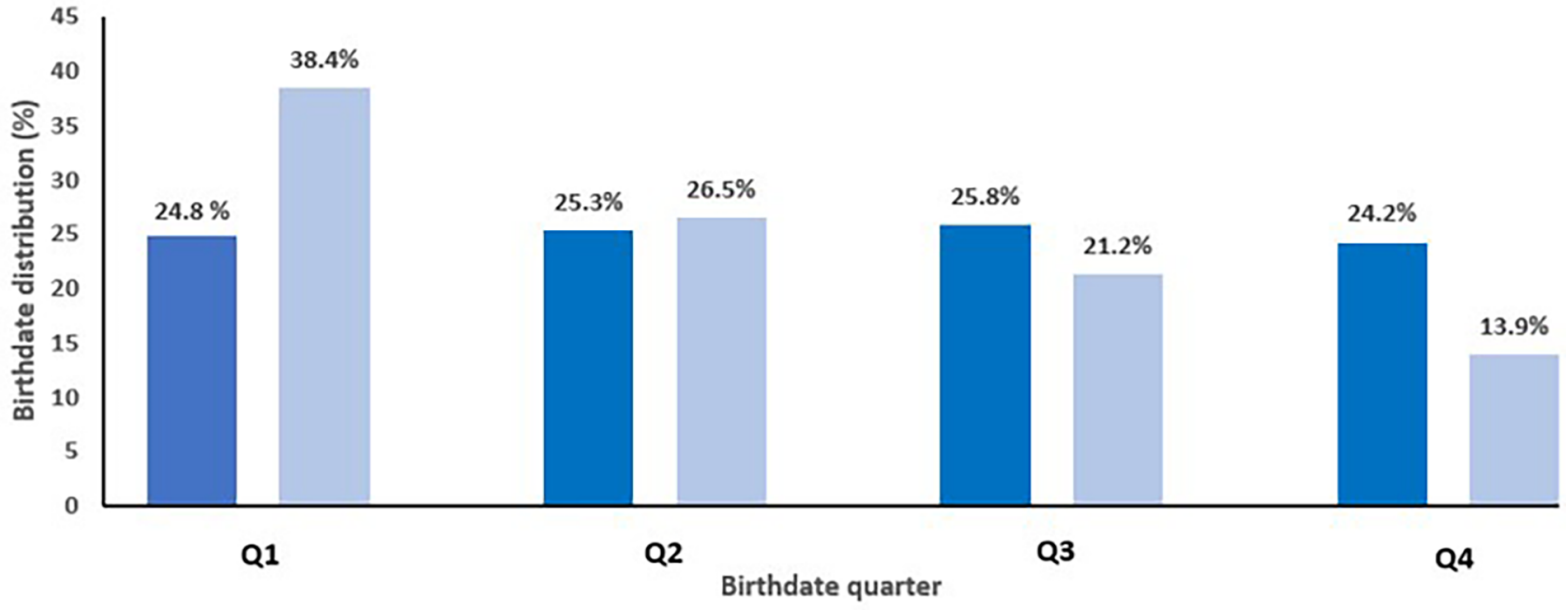
The expected distribution of the Belgian population per birthdate quarter (1990–1996) (dark blue bars) versus the observed distribution of soccer players per birthdate quarter (light blue bars) of the whole database.

The age distribution of our study population showed a RAE for the overall dataset (Figure 1). Figure 1 shows that there was a significant difference in the distribution of players according to a birth quarter (*p* < 0.01). There was a dominance of players that were born in Q1 (38.4%), followed by players born in Q2 (26.5%), Q3 (21.2%), and Q4 (13.9%). The RAE was also clearly present in all the different individual age categories (1990–1996). The magnitude of the RAE was 2.61 (OR: 2.61; 95%CI: 1.14–5.98) for the overall sample of youth players (Q1 = 38.4% vs. Q4 = 13.9%), which can be correlated with a medium effect size. An uneven distribution was identified for each annual group, with 31.2%–50.0% of players born in Q1 and 4.5%–21.9% in Q4. %. The distribution of the soccer players, on the other hand, showed a statistically significant difference between the percentage of observed soccer players from the first quartile vs. the percentage of the last quartile, respectively 38.4% (58/151) vs. 13.9% (21/151) (Figure 1).

These asymmetric birthdate distributions, presenting the RAE, were statistically significant: Chi-square method (*χ*^2^ = 19.46, *p* < 0.01) and the Kolmogorov-Smirnov method (*p* < 0.01). The correlation coefficient (*r* = −0.85, *p* < 0.01) also showed a statistically significant decreasing trend in the number of players from January to December.

In Figure 1, the expected distribution of the Belgian population per birthdate quartile (1990–1996) was plotted vs. this observed distribution of the 151 soccer players. The expected Belgian population was equally distributed per quartile (range 24.2–25.8).

### RAE and personality constructs

**Figure 2:**
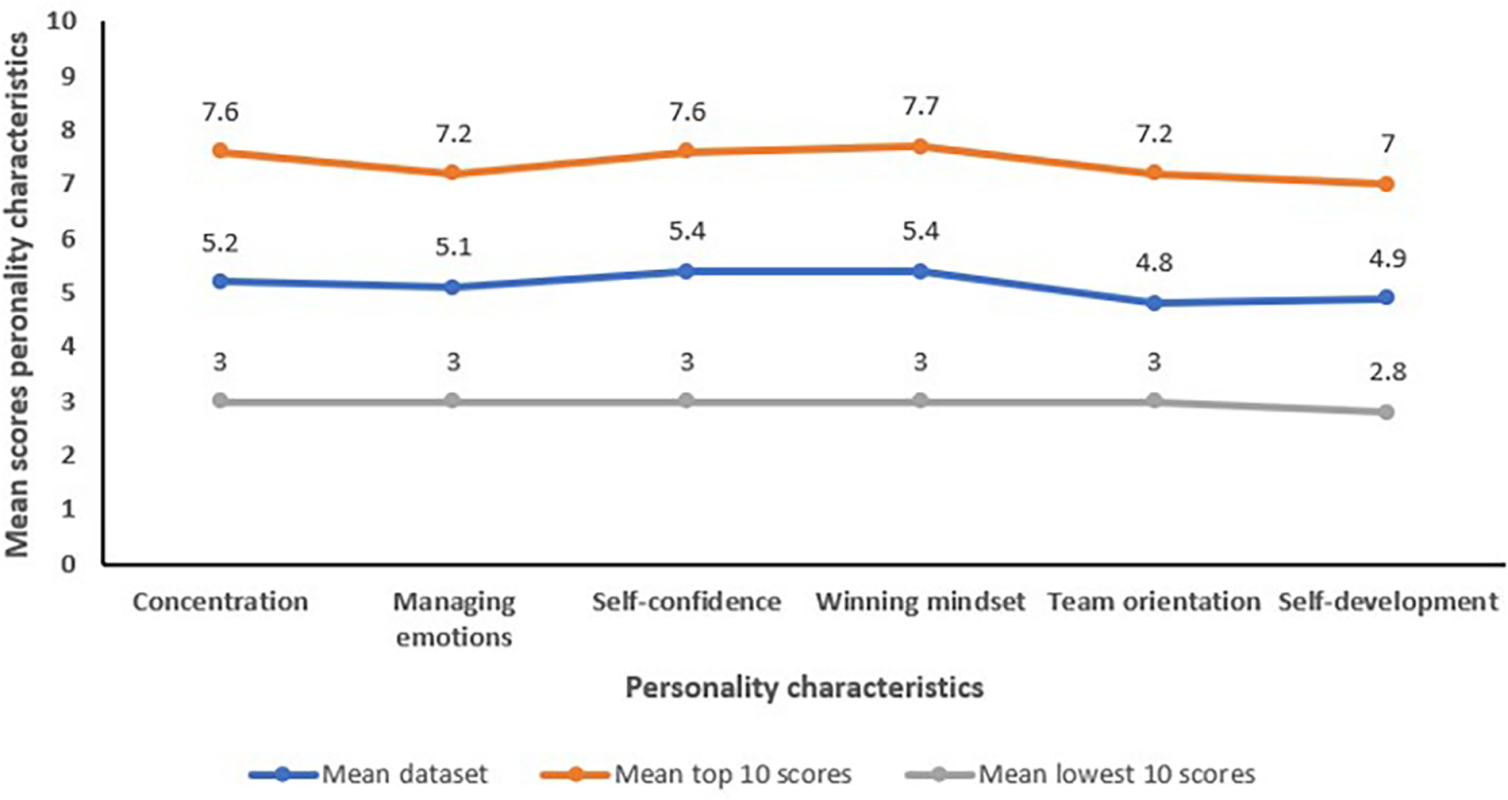
Combined data of the observations, questionnaires and interviews were used to score the youth soccer players on the six personality constructs (concentration, managing emotions, self-confidence, winning mindset, team orientation, self-development) on a nine-point standard scale. Mean scores for the whole database, for the top 10 highest scores and lowest scores for every personality construct are assessed.

The highest mean scores and mean top 10 scores over the whole dataset were reached on personality constructs like self-confidence (mean 5.4, mean top 10 scores: 7.6) and winning mindset (mean 5.4, mean top 10 scores: 7.7) by the elite youth Belgian players (Figure 2). The lowest mean scores and lowest top 10 scores were reached for personality constructs like team orientation (mean 4.8, mean lowest 10 3) and self-development (mean 4.9, mean lowest 10 2.8) (Figure 2).

The median scores for personality constructs and the corresponding *p*-value for the first vs. last quartile are shown in Table 1. The only personality construct with a significantly higher median score for the players from quartile 4 (score 6) compared with quartile 1 (score 5), which was statistically significant (*p* = 0.04), was self-confidence. Players of the last quartile also had higher median scores for personal constructs such as concentration (6 vs. 5, *p* = 0.34) and winning mindset (5.2 vs. 5, *p* = 0.23) (Table 1).

**Table 1:** Median scores of the players on personality constructs on a nine-point standard scale, based on observations, interviews and questionnaires pro birthdate quarter (Q) with consideration of statistical significance by comparing median scores of Q1 with median scores of Q4 (*p*-value).

	*p*-value	Median score pro quarter			
		(Q) Q1	Q2	Q3	Q4
		*n* = 58	*n* = 40	*n* = 32	*n* = 21
Self-confidence	0.04*	5	5	5	6
Winning mindset	0.23	5	5	5	5.2
Self-development	0.45	5	5	5	5
Managing emotions	0.12	5	5	5	5
Concentration	0.34	5	5	5	6
Team orientation	0.41	5	5	5	4
Total	**0.12**	**5**	**5**	**5**	**5.2**

* *p* < 0.05 (statistically significance); Q, birthdate quarter.

The bold values provided are the mean scores and *p*-values of the players on the sum total of personality constructs pro birthdate quarter.

Table 2 shows the median scores for personality constructs and the corresponding *p*-value for both semesters. There was a non-significant higher median in semester 2 (score 6) compared with semester 1 (score 5) for “self-confidence” (*p* = 0.06). This result was approaching statistical significance (*p* = 0.06). For the personality construct “team orientation”, the median was significantly higher (*p* = 0.03) in semester 1 (score 5) compared with semester 2 (score 4).

**Table 2:** Median scores of the players on personality constructs on a nine-point standard scale, based on observations, interviews and questionnaires pro semester (S) with consideration of statistical significance by comparing median scores of S1 (born between January and June) with median scores of S2 (born between July and December) (*p*-value).

	*p*-value	Median score pro semester (S)	
		S1	S2
		*n* = 98	*n* = 53
Self-confidence	0.06	5	6
Winning mindset	0.38	5	5
Self-development	0.31	5	4.5
Managing emotions	0.31	5	5
Concentration	0.19	5	5
Team orientation	0.03*	5	4
Total	**0.22**	**5**	**5**

* *p* < 0.05 (statistically significance); S, semester.

The bold values provided are the mean scores and *p*-values of the players on the sum total of personality constructs pro semester.

### RAE and long-term career evolution

**Figure 3:**
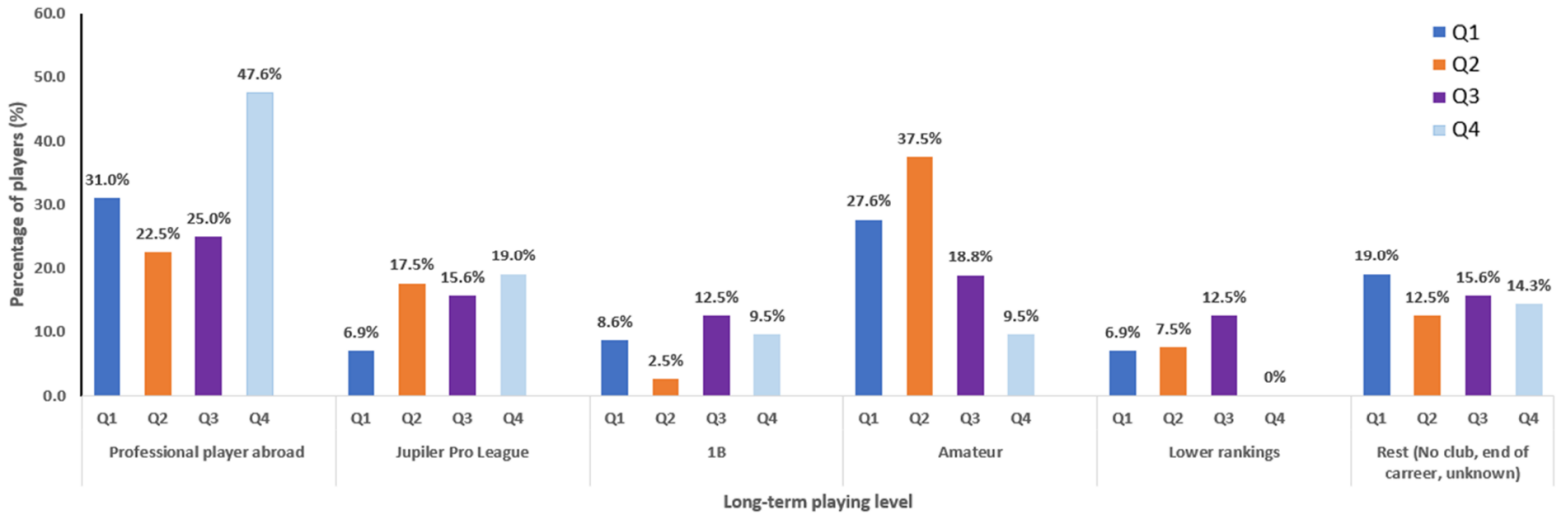
Evaluation of the long-term career development by assessment of the long-term playing level of the whole database per birthdate quarter (Q): players were categorized as professional player abroad, first division Jupiler Pro League, second division 1B, Amateur player, player in lower ranking and player who ended their career (rest).

The mean FU of the study population was 8 years. Figure 3 shows the players’ long-term playing level per quartile. 76.2% (16/21) of the players born in the last quartile were contracted as professional soccer players (abroad, Jupiler Pro League, or 1B). For the first quartile, only 46.6% of the players (27/58) had a similar professional status (Figure 3).

Of the players born in the last quartile, compared with the players born in the first quartile, more players were signed as a professional soccer player abroad (Q4 47.6% vs. Q1 31.0%), as a professional soccer player in the first division Jupiler Pro League (Q4 19.0% vs. Q1 6.9%) and as a professional soccer player in the second division 1B (Q4 9.5% vs. Q1 8.6%) (Figure 3).

A non-significant higher number of players born in the first quartile ended their career or didn't find a club (11/58, 19.0%) later, compared with players born in the last quartile (3/21, 14.3%).

**Figure 4:**
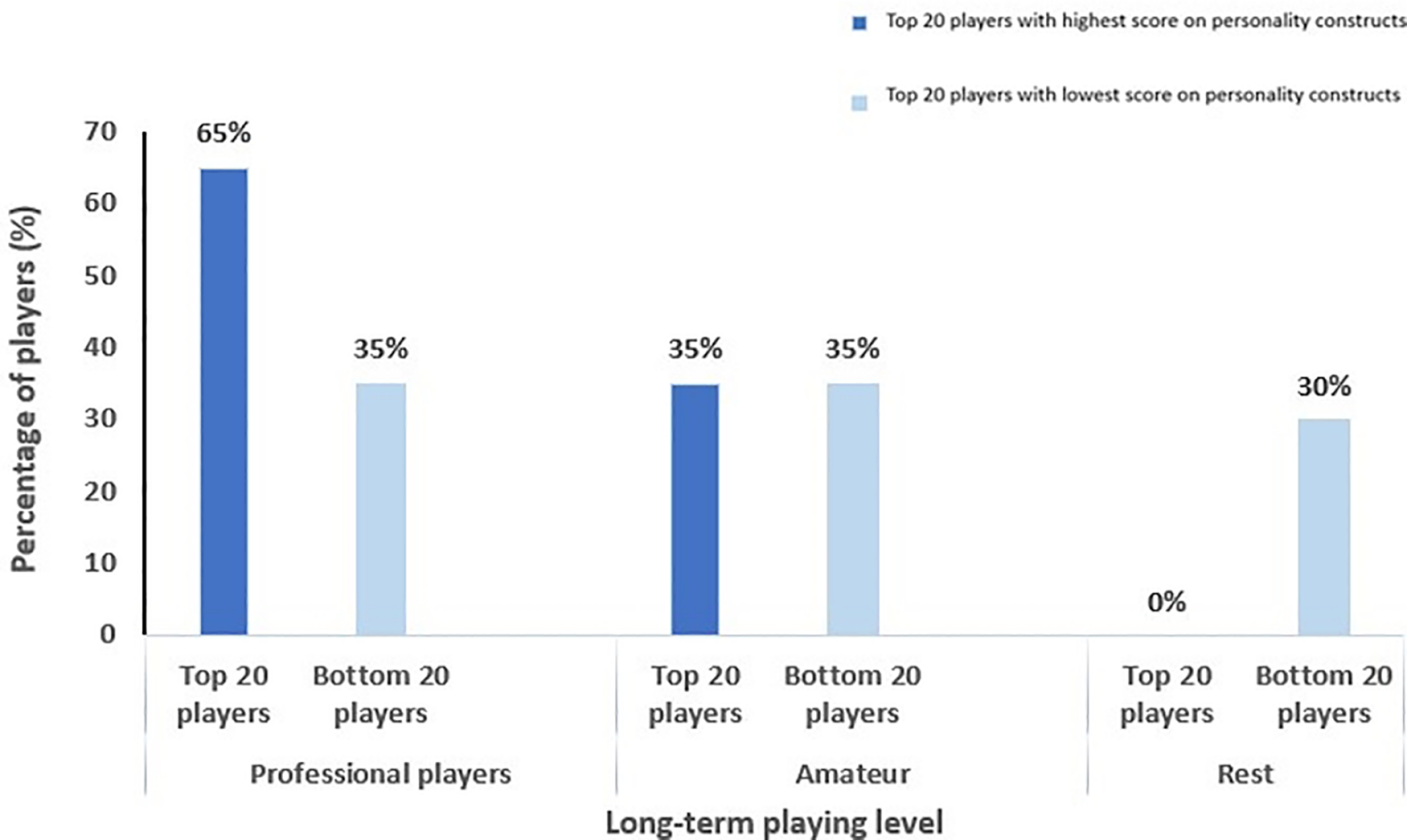
Correlation between long-term playing level and scores on personality constructs. The 20 players with the highest scores (dark blue bars) on personality constructs were compared with the 20 players with the lowest scores (light blue bars) on personality constructs.

Of the 20 soccer players who had the highest total score on personality constructs, 13 players (65.0%) received a professional contract. This was in contrast with the 20 lowest scoring players, of which only 7 (35.0%) were professionals (Figure 4).

Of the 20 soccer players who had the lowest total score on the personality constructs, more (6/20, 30%) players finished their career or were not able to sign with a club, compared with the 20 soccer players who had the highest total score on the personality characteristics (0/20, 0%) (Figure 4). More positive scores on psychological constructs appears to scaffold one's later success in professional soccer.

### Multiple stepwise regression analysis

A multiple stepwise regression analysis was carried out to investigate which parameters are critical to predict professional future performance.

Birth date quarter, future professional level and the 6 personality constructs were implemented into forward stepwise multiple regression analysis. The aim would be to identify which parameters would predict professional success best and add independently the most in predicting future professional level (Multiple R) by taking out the covariation.

The results of the regression indicated that the model of predicting future professional level explained 37.3% of the variance and that the model was a significant predictor of future professional level [*F* (8, 148) = 2.87; *p* = 0.05]. Self-confidence contributed most to future professional level (regression coefficient 4.77; *p* = 0.60) comparing with the other 5 distinct personality constructs and birth date quarter (regression coefficient 0.10; *p* = 0.43). These predictors were not significant.

In case of restricting possible predictors for future professional performance to quarter and self-confidence, a correlation of 32.9% between future professional outcome, birth date quarter and self-confidence could be observed [*F* (2, 148) = 8.98; *p* < 0.05]. Self-confidence contributed most to future professional level. While self-confidence contributed significantly to prediction of professional level (regression coefficient 0.397; *p* = 0.12), quarter did not (regression coefficient 0.148; *p* = 0.27).

In case of predicting future performance level, lonely based on birth date quarter in advance of late mature players; a correlation of 12.8% between both parameters could be observed [*F* (1, 149) = 2.49; *p* = 0.12]; in which quarter was not significant.

On the other hand, by predicting future performance outcome, lonely based on self-confidence; a correlation of 31.7% can be identified [*F* (1, 149); *p* = 0.00007] (Table 3). All the other factors contribute each independently less than 1% of R square change.

**Table 3:** Multiple stepwise regression analysis is presenting what each individual parameter adds independently in terms of predicting who attains professional level.

				95% CI Regr. Coeff		
Parameters	Mult. R (%)	R sq.(%)	Regr. coeff.	Low. 95%	Up. 95%	*p*-Val.
Self-Confidence	31.70	10.10	0.24	0.13	0.36	0.00007
Quarter	12.80	1.60	0.08	−0.02	0.17	0.12
Total Characteristics	10.30	1.10	0.04	−0.02	0.11	0.21
Semester	10.0	1.0	0.03	−0.02	0.07	0.22
Winning mentality	9.80	0.95	0.07	−0.04	0.18	0.23
Team orientation	9.10	0.82	−0.06	−0.17	0.05	0.27
Managing emotions	8.30	0.68	0.06	−0.06	0.18	0.31
Concentration	5.80	0.33	−0.04	−0.17	0.08	0.48
Self-development	1.80	0.03	−0.01	−0.12	0.1	0.82

Total characteristics implicate the sum of all the personality constructs together (Self-confidence, winning mentality, team orientation, managing emotions, concentration, self-development). Following the multiple stepwise regression analysis, self-confidence (Multiple R of 31.70%) contributes most concerning the prediction of future professional level.

Mult. R, Multiple R; R sq., R square; Regr. coeff., Regression coefficient; Low. 95%, Lower 95%; Up. 95%, Upper 95%; *p*-Val., *p*-Value.

So, following the multiple stepwise regression model; there is a co-linearity across personality characteristics and relative age effects, in which the personality characteristics of self-confidence contributes most (multiple *R* = 31.7%, R square = 10.1%, *p* = 0.00007), concerning the prediction of future professional outcome.

## Discussion

The RAE is a well-researched phenomenon in soccer (19, 44–46). In childhood and adolescence, youth football players are categorized by chronological annual age groups, driven by the arbitrary “cut-off” or “selection” dates (5). Both, within sport and educational contexts, individuals are often divided into chronological age categories in an attempt to ensure fairness and equality. However, the chronological age gap of up to 12 months between players born early (January) and late (December) in the year leads to substantial variation in physical performance and finally biased talent selection decisions (6, 7, 9, 19). The 151 players in this study were grouped within categories according to typical Belgian domestic soccer season birthdate quartiles depending on their date of birth and expressed as a percentage of the sample population with a cut-off date of January 1st. The result of participation or selection bias because of maturity-related bias, specifically the overrepresentation of chronologically older soccer players within one age category, is called relative age effects (RAEs), which describe the (dis)advantages associated with being the relatively youngest or oldest within a particular age category (13). RAEs effect talent development systems and academies in a wide range of team and individual sports, e.g., ice hockey, soccer, swimming, and tennis, in both female and male categories from 4 years of age to adulthood (8, 11, 15, 45, 47). The age distribution of our study population showed a clear RAE for the overall dataset. Relative age and maturity selection bias can both confound academy soccer talent selection and development strategies (6, 48). Relatively old children within chronological annual age categories are more likely to be selected in talent development teams, with selection accompanied by additional training, and access to higher quality coaching with better opponents, likely leading to accumulated performance advantages (4, 6, 9, 19, 24).

As stated earlier, these early maturing players are often marked as possessing temporary, maturity-related advantages in anthropometric (e.g., stature and body mass) and physical fitness characteristics (e.g., power, strength, speed) (16, 18). These temporary advanced somatic characteristics are often perceived as dominant by talent scouts and coaches, because they typically characterize key tactical roles and playing positions (1, 2, 4). Subsequently, this can lead to a (sub)conscious reduction in selection opportunities or abandonment of relatively young players (6, 19).

So in consequence, the relatively young players, who may also be smaller and less physically developed, but who have equal technical and tactical ability, are underrepresented. As a consequence they are less likely to be selected for talent development systems and academies, and are finally more likely to withdraw early from sports (19). This biased selection during youth talent development programs in soccer academies limits a relatively young player's chances of succeeding later in their career transition to a professional player. Of the 20 soccer players who had the lowest total score on the personality constructs in our study, more players (30%) finished their career or were not able to sign with a club, compared with the 20 soccer players who had the highest total score on the personality characteristics (0%). The late-maturing players may suffer other consequences in academies as they are disadvantaged by lower selection quotas by scouts, which may lead to less competition experience and exposure to better opponents. This may cascade to lower motivation and less access and exposure to high-quality training. However, there is a ray of hope because recent RAE studies have found that by reducing RAE in talent development programs, there are benefits to these same players as they age. This suggests there are delayed benefits if late maturing players can be sustained within the talent development system (11).

Children continue to learn, mature and develop in all ways as they get older. Under existing youth systems, they are grouped according to their chronological age groups in educational and sports settings. The primary aim is to allow them to develop and compete with comparable individuals to ensure fairness and account for maturational differences and, as such, to give them all the same opportunity for sports participation and success in talent development (5). To set objective limitations and provide developmentally equitable competition, these chronological categories are bound by “cut-off” dates. In the Flemish educational and football system, pupils are organised into one-year age groups, using January 1 as the cut-off date. We remarked a nearly threefold overrepresentation for youth players born in the first quartile of the selection year as well as a clear underrepresentation of players born in the last quartile in our study. All this happens in spite of the fact that, maturational effects may result in large development inequalities between chronological and biological age (48). This is particularly true throughout puberty. However, in any given educational or sporting year-group, this chronological birthdate distribution has significant consequences for future successful performance by its consequential impact on talent selection and progression, known as the relative age effect (RAE) (6, 12, 49, 50).

Interestingly, previous research has illustrated how relatively younger players, who are selected for a talent development system, actually have in the long-term a greater chance of becoming a professional player than their initially relatively older opponents (11, 30). These observations have become encompassed in the “underdog hypothesis” (11, 37). Late-maturing players are more likely to be signed professionally and reach prestigious career during their professional development. Concerning the players’ long-term playing level per quartile in our study, 76.2% of the players born in the last quartile were contracted as professional soccer players, in comparison with only 46.6% of the players with a similar professional status for the first quartile. This is because of multiple factors; for example they may benefit more from competitive play and trainings with their older opponents after selection, or it may be because of their stronger psychological factors (2, 27, 37). Surely there are organizational and financial benefits for clubs if soccer academies can accurately and efficiently recruit and develop “home-grown” potential in their youth players (1, 51). Likewise clubs recognize that individually highly skilled players usually result in better team performance, and the route to more talented, highly skilled individual players is through effective youth academies and training. Research that provides greater insight into the causative processes and mechanisms of the RAE and personality structure, and therefore more effective talent identification, may lead to better future performance productivity and talented players for clubs (1, 2). From an economic perspective, it is often more cost effective for football clubs to buy undervalued players with initial lower market values. Lower market values may be initial assessed because of lower physical constructs. Clubs hope for higher performance and higher returns on investment based on player's perceived strong psychological profile. Therefore, assessing the actual and future values of possibly talented youth soccer players requires a multifactorial approach with physical, technical, tactical and psychological assessments amassed to assist talent identification and training profiles for each individual player.

This study showed that late-mature players have higher-value senior professional contracts in the long-term. Romann et al. revealed that late-mature players have higher market values over time as well and are undervalued in younger age groups (52). Our belief is that current as well as future players’ real market values are based on both their physical and psychological characteristics and will, therefore, play an increasing role in talent recruitment, sports economics and talent development for clubs. Allof these factors dramatically impact sport development academies in their role as the primary entrance point for most professional talented players.

The first objective of this study was to identify the presence of a significant RAE in this cohort of selected Belgian elite youth players. The second objective was to assess the association between RAE and personality constructs by selected elite youth players. The third objective was to assess the association between the long-term career development of a senior soccer player and their initial personality factors.

Our results clearly show that the RAE is present for the whole database and each age category (Figure 1). Relative age distributions between quartiles for the whole database are shown in Figure 1.

In this cohort of 151 Belgian youth elite players, the magnitude of the RAE was a factor of 2.61 (OR: 2.61; 95%CI: 1.14–5.98, medium effect size) for the overall sample of youth players (Q1 = 38.4% vs. Q4 = 13.9%) (Figure 1). A significant uneven distribution was observed for each age group (1990–1996), with 31.2%–50.0% of players born in Q1 and 4.5%–21.9% in Q4. So, we saw a nearly threefold overrepresentation for youth players born in the first quartile of the selection year as well as a clear underrepresentation of players born in the last quartile. This typical distribution of the RAE of squads has also been reported in earlier studies (9, 16, 18). Our findings illustrate that for the entire cohort of 151 Belgian youth elite players, relatively old and more mature players (quartile 1) were 2.61 times more likely to be registered and participate in development elite programs compared to the quartile four relatively young and late-maturing players. These odds ratios of RAE were comparable with those determined prior in Belgian soccer academies.

We observed that selected players from the U16 to U19 age categories demonstrated equal anthropometric and fitness phenotypes. Only relatively young players with advanced normative growth and maturation, were selected to receive advanced coaching opportunities and education in training and competition.

Previously, a developmental systems model was used to explain the mechanisms of the RAE (25, 46, 49). Wattie et al. suggested that the RAE in sports is based on individual, task, and environmental constraints (46). These effects are augmented by a more favorable alignment between the characteristics of relatively old youth and the demands of their developmental environment. As an individual constraint, the physical maturation of an athlete is important. More physically mature individuals have greater chances to be selected by coaches and talent scouts and exhibit different levels of ability and potential (1, 2). This is an advantage for relatively old athletes because physical development, namely stature and body mass, follow chronological age. It should be noted that relatively young athletes who have matured early are also provided more opportunities to be selected because of their physical characteristics (6, 17, 24). Gender can be seen as another individual constraint. Although the RAE is also observed in female athletes, it has been founded to occur less consistently and with a smaller magnitude (12, 14). It is hypothesized that this smaller RAE could be caused by less required competition among female athletes to gain access to an elite team (8). Social pressures that encourage adolescents to conform to gender-based stereotypes perhaps discourage females from participating on a more competitive level, especially early-maturing females. This can result in a smaller RAE.

Although maturity-related factors are essential components of RAEs, they are also underpinned by other, more global factors, which can influence the developmental contexts of the players (7, 24, 46).

Task constraints are relevant factors and refer to sport type and level of competitive play (46, 53). Sports, where physical characteristics are important, are more likely to favor athletes with advanced physical development, which is a possible benefit to relatively old youth players. An increase in RAEs with increasing competition levels has been identified (14, 54, 55). The more players competing for a finite number of places on teams, the more likely that the characteristics of relatively old youth may appear to optimally align with environmental and task demands, and the stronger the size of the effect.

Environmental constraints refer to the popularity, categorizing of the sport, policies governing its play, and family influences (46). So, for example no systematic RAE has been noticed in American football, which possibly can be explained by the fact that some American football leagues use a sub-classification based not only on age but also on body mass. All those individual, task and environmental constraints should not be seen as separate entities, because they all interact.

However, as has been demonstrated elsewhere and as we have seen in this research, components of the RAE are multifactorial (2, 14, 25, 46, 49). Thus, the impact on psychological and cognitive parameters needs to be evaluated, as psychological factors are considered important by practitioners during the talent selection process (1, 2, 4). Although the development of physical performance is a crucial element of talent identification, all other areas of performance (technical, tactical, psychological, or cultural) should be considered as objective selection criteria necessary to properly evaluate a player's quality and long-term development to improve talent identification.

Within professional youth soccer academies, it is the objective to promote the physical and technical skill development as well as personality development of talented players, which reflects the belief that personality plays a crucial role in players’ future success (2, 27). Psychological characteristics have elsewhere been integrated into models of talent identification and development as significant predictors of success in sports and general evaluation of players (12).

Musch and Grondin explain and integrate the psychological factors that underlie the RAE in greater depth through various social agents (12). The way in which these social agents (parents, coaches, and athletes) interpret mechanisms such as physical stature, maturity, and cognitive ability creates RAEs through various effects such as the Pygmalion, Galatea, and Matthew effects (12).

Children who are relatively old are more likely to be perceived as more talented by their peers, families, and coaches. Temporary, physical fitness, and anthropometric advantages afforded to older and more mature players are often the reason why relatively old players are considered more talented (19, 46). The Pygmalion effect refers to the perception that the greater the expectation placed on an individual, the greater the result that individual will attain. These environmental influences can lead to better performance.

The Galatea effect is comparable, but here the athletes’ expectations of themselves enhance their willingness to perform (25). The Galatea effect is a second form of self-fulfilling prophecy, whereby once expectations are placed upon an individual, that individual typically acts congruently with those expectations.

A third psychological and sociological effect related to the RAE is the Matthew effect: individuals who start with an advantage are more likely to keep their advantage over time (25). Specifically, relatively old children have greater access to advantages than their relatively young peers.

The first purpose of this study was to examine if there is any relationship between the RAE and personality constructs in elite Belgian youth soccer players. Personality and psychological factors are important in sports because they may influence an athlete's performance subcomponents (such as technical, tactical, and physical skills), and eventually, success in competition and talent selection (1, 2, 4, 27). Understanding an athlete's psychological profile can be useful for designing both general psychological training and interventions specific to the sport, position, and role of the player on a team (1, 4, 27, 28, 43, 56). This theoretical framework can be used to further understand the RAEs and eventually used to create policies aimed at limiting the negative effect of relative age in soccer and creating more equal opportunities for all players. In our study, we have focused on self-confidence, winning mindset, self-development, managing emotions, concentration, and team orientation.

Results from this study show that the highest mean scores and mean top 10 scores over the whole dataset were reached on personality constructs like self-confidence and winning mindset by the elite youth Belgian players (Figure 2). The lowest mean scores and lowest top 10 scores were reached for personality characteristics like team orientation and self-development (Figure 2). Therefore, personality characteristics like self-confidence and a winning mindset are important values to qualify and be selected as elite youth soccer players.

Among young elite players self-confidence is paramount to their future success as a professional player. Level of self-confidence and RAE together account for approximately 32% of one's success at the professional level.

Within professional youth soccer academies, it is obviously important to promote physical and technical skill development as well as personality and psychological development of talented youth players, which reflects the stance that personality plays an important role in players’ future success and development as senior professional players (57). Elsewhere psychological constructs and development have been integrated into models and procedures of talent identification and development. psychological constructs have also been identified “as significant predictors of success” in sports (32, 58). Thankfully because elite youth academies require clubs to focus on both psychological development and support of players, academies have started to include psychological constructs in their general evaluation of players (27). Therefore, in recent years scouting sheets assessing and evaluating technical and tactical skills have been revised to include and evaluate these psychological constructs. Youth elite academies ask their staff regularly to evaluate the players’ psychological constructs and development. Previous research has confirmed that sport-specific self-report questionnaires revealed many different psychological constructs, ranging from motivational aspects (27) to self-regulation (22) or the use of coping strategies (59). Earlier studies concluded that self-regulation, resilience, commitment, and discipline had an important impact on player development and future success (60). Previous studies also stated that successful youth players scored better on psychological constructs like motivation, confidence, self-referential cognitions, and emotion by applying a questionnaire (35). Furthermore, strong psychological constructs were positively associated with current and future soccer performance and development (27, 35, 58). To our knowledge this is the first study using multiple regression to demonstrate the relative strength and importance of one's self-confidence level related to RAE.

In our cohort of elite youth players, the players born in the last quartile had the highest median score on self-confidence (6 vs. 5), which was statistically significant (*p* = 0.04) (Table 1). Players in the last quartile also had higher median scores for personal constructs such as concentration (6 vs. 5, *p* = 0.34) and winning mindset (5.2 vs. 5, *p* = 0.23), but these results were not statistically significant (Table 1). There are multiple potential mechanisms to explain the observed relationship between RAE and personality constructs. Firstly, this is in line with the hypothesis that a possible cause of the RAE may be the Galatea effect. They have “beaten the odds” by being selected despite being relatively younger. Thus, the players born in the last quartile perceive themselves as more skilled. This results in a better work ethic which makes them better players and enhances their self-confidence (25). Galatea effects are a possible theory to explain the curious phenomenon of relatively young players excelling at professional levels. Possibly, relatively young or late-maturing children who manage in the end to be selected to elite youth teams also have increased self-expectations and motivation, which, after years of training, lead to professional success and development in youth academies (11, 22). So, we can connect the RAE with the Galatea effect.

Secondly, in case late-maturing players are to be selected for elite soccer academies, then they would take advantage to possess and/or develop more adaptive psychological attributes and skills. Concerning the underdog hypothesis, late maturing players may also need to develop more adaptive and efficient self-regulatory skills if they are to remain competitive within their age groups and would be selected for competitions and training. Former studies showed that delayed maturation was associated with greater self-regulation, planning, reflection, and evaluation. These more adaptive and efficient learning skills and psychological strength may help overcome some of the physical and functional disadvantages associated with later maturation (e.g., smaller stature, inferior strength, speed, power).

In the cohort of elite youth players, players born in the first semester scored better on team orientation, which was statistically significant (*p* = 0.03) (Table 2). Again, we observed that players born in the second semester, scored higher on self-confidence, compared with players of the first semester (6 vs. 5), in which the median scores were near statistical significance (*p* = 0.06) (Table 2). Concerning the observation that relatively old elite players scored better on team orientation, the Galatea effect could also play a crucial role. From a young age, relatively old players are more likely to get selected for an elite team. This causes them to see themselves as more competent. Galatea effects might provide a theory that explains long-term athletic attainment (25). Specifically, it is often stated that athletes are selected for elite teams based on physical maturity rather than skill (61). This would create false self-beliefs from relatively old and more mature athletes concerning their sports skills, which would cause the initiation of the Galatea effect. Next, as athletes are convinced of those expectations, they raise their self-expectations of abilities and behaviors that match self-expectations and motivation, affording continued success and selection in elite soccer academies (25). These new behaviors might include more diligent and frequent training sessions, which would suggest the Galatea effect. So, we can connect the RAE with the Galatea effect.

This effect is also enhanced by the Pygmalion effect, in which coaches and parents reinforce this competence. The Pygmalion effect refers to the inherent perception that the greater the expectation placed on an individual, the greater the result that the individual will attain in the end (25). Conversely, when lower expectations are placed upon individuals, outcomes will be inferior. Coaches set expectations on athletes and make selections and determinations of talent based on physical maturity (62–64) rather than multifactorial skill or potential. Therefore, Pygmalion effects falsely based on physical maturity might lead to higher expectations for relatively old and early-mature children, elucidating why relatively old players succeed. Pygmalion effects are typically initiated from power relationships, such as teacher-pupil or employer-employee. In sport, the most appropriate relationship to situate the Pygmalion effect is the coach-athlete relationship, which will explain unequal selections (25). In sports like soccer, coaches will have expectations for athletes; however, expectations based on false beliefs might perpetuate unfair advantage. These effects are even apparent after selection (25). Coaches often orchestrate practices in groups and interact with players during competitions and training, in which they treat players differently, possibly founded on inherent expectations. Consequently, youth soccer coaches offered high-expectancy players more reinforcement, while low-expectancy players received more general instructions. High-expectancy players are awarded more supplementary feedback, praise, and efficient instruction compared to low-expectancy players (25). Perceived competence is one of the characteristics related to peer leadership behavior. Players with peer leadership behaviors contribute to social cohesion and team efficacy (28).

Coaches and parents facilitate Pygmalion effects in soccer (25). Expectations of coaches and scouts might perpetuate or amplify RAEs that are initiated at earlier ages by parents and teachers. The impact of social agents on RAEs is crucial, more specifically the inherent effect of the Pygmalion and Galatea frameworks on RAEs. Possibly, if Pygmalion and Galatea effects of parents and coaches on players can be reduced, RAEs might also decrease, thereby helping to create a non-discriminatory soccer setting.

The existence of RAEs causes inequal distributions (5, 9, 19, 45). Fundamentally, asymmetric distributions are acceptable in soccer, but we believe they should be based on skill, talent, and potential rather than a birthdate, stature, or maturity. Social agents like parents and coaches have a strong impact on RAE by Galatea and Pygmalion effects. Nevertheless, it appears that social agents sometimes interpret physical maturity as talent, in terms of stature and body mass (63). Matthew, Pygmalion, and Galatea effects are intrinsically integrated and implemented by parents, coaches, and players as they relate to the RAE. Coach selections related to relative age would also be influenced by parental enrolment decisions (25). The influence of players on the RAE is determined due to Galatea effects, or the self-expectations that players possess, which might be higher for relatively older athletes. The higher expectation of players would be caused by the influence to which players indirectly are exposed by coaches and parents. Therefore, coaches and parents exert their influence and impact on RAEs indirectly through the self-expectations of players (25). For coaches, we also noted a clear reciprocal Pygmalion effect (25). Coaches tended to place higher expectations on relatively old players, which might also reflect the RAEs. Therefore, when relatively old players fulfill these higher coach expectations, they complete the self-fulfilling prophecy, which in turn would cause coaches to further increase expectations of their players, hence a reciprocal process. Alternatively, a possible effect of coaches placing high expectations on relatively old team players is that parents also share these higher expectations. Therefore, parents might increase the self-fulfilling prophecy and RAEs by placing higher expectations on their relatively old children. It may be hypothesized that by eliminating Matthew effects and Pygmalion effects, RAEs could also be minimized, or at least influenced, but further research is necessary. By getting more information about the working mechanism of the Pygmalion and galatea effect, a deeper understanding of the impact of RAEs would be gained, which might lead to concrete proposals that could reduce RAEs in soccer and create equal opportunities for all participants.

Personality characteristics like self-development and managing emotions showed no difference between players born in the first or last quartile (Table 1). This can be explained by the selected cohort of elite youth soccer players, so by the fact that these players were already preselected. Elite youth soccer players are more likely to own better self-regulatory skills like self-development (22).

In case late-maturing players are to be selected for elite soccer academies, then they would take advantage to possess and/or develop more adaptive psychological attributes and skills (4, 22, 35). Self-regulation is the process whereby a player takes ownership of their development by establishing personal goals, controlling their feeling, engaging in action to achieve these goals, including self-initiated processes to convert mental abilities into physical skills in the learning process and by evaluating their progress (22, 29). Players who excel in self-regulating also approach tasks with a high level of effort and possess increased levels of self-efficacy in managing general task situations (29). Self-regulation has been a key personal construct in youth elite soccer to manage effective learning, develop future potential, and differentiate between successful and less successful future professional senior players (22). Players who excel in self-regulated learning have been shown to use effective planning to improve their daily performance, evaluate training outcomes, and reflect on their development processes if learning objectives and strategies have been achieved with consideration of strengths and weaknesses (22). Elite youth soccer players possess more adaptive self-regulation than non-elite players, suggesting that self-regulation contributes to successful professional development as an elite youth soccer player (22). Higher levels of self-reflection and effort were identified by elite youth players. They appeared more willing and efficient to invest effort into successful task execution and were capable of adapting their knowledge and actions in order to execute skills (22). Failure to manage self-regulated learning has been shown to negatively impact performance outcomes in soccer (38). Concerning the underdog hypothesis, later maturing players may also need to develop more adaptive and efficient self-regulatory skills if they are to remain competitive within their age groups and would be selected for competitions and training. Former studies showed that delayed maturation was associated with greater self-regulation, planning, reflection, and evaluation (11, 22, 30). It is important to note that in earlier studies no correlation has been identified between relative age, adaptive self-regulative learning, planning, reflection, evaluation, and managing emotions (37). That may be an explanation for why no differences in median scores were observed in this study for personality constructs like self-development and managing emotions (Tables 1, 2). Rather, it can be suggested that relative age and maturity selection biases exist and operate independently in elite soccer academies (48).

In correlation with the underdog hypothesis, more adaptive engagement in self-regulated learning, in particular, self-evaluation and reflection were identified by late-maturing players (37). These more adaptive and efficient learning skills and psychological strength may help overcome some of the physical and functional disadvantages associated with later maturation (e.g., smaller stature, inferior strength, speed, power) (65). These late-maturing players may have an advantage because of a stronger psychological profile as a senior professional player, when maturity-associated differences in stature and function have attenuated or, in some cases, reversed. This psychological advantage will only be realized, however, if later maturing players are selected into and retained within the elite youth soccer academies to gain experience in qualitative competitions and training. An isolated more adaptive self-regulation profile, though desirable in the long-term development of elite youth soccer players, may not be sufficient to overcome the physical disadvantages associated with later maturation and/or guarantee progression to the most senior professional levels (22, 37). In support of this contention, equal scores in self-regulation were revealed in both relatively younger and older players (37). Thus, further strategies are required to ensure that talented, yet less mature, soccer academy players are not overlooked and excluded from elite youth soccer academies. It should be noted, however, that the players in the current study represent a highly select group of elite youth Belgian soccer players. Differences in relative age may exert greater influence upon self-regulated behavior by a broader cohort of different playing levels and younger ages. It is difficult to state whether later maturing players had always possessed more adaptive self-regulatory skills or if they developed as a result of the greater challenges that they had faced because of smaller physical stature. Thus, greater understanding is needed on how self-regulatory skills develop and the role that they play in the processes of selection and retention in elite youth soccer academies.

Finally, the current playing level of the study population as senior players was considered, approximately eight years after the initial data collection to assess which players reached the senior elite professional level. Results showed that players born in the last quartile were more successful compared to players of the first quartile (Figure 3). 76.2% of the players born in the last quarter were contracted as professional soccer players at the senior level, compared to 46.6% of the players born in the first quarter. More players of the last quarter received a professional contract as senior elite players at all the different professional playing levels: professional player abroad (Q4 47.6% vs. Q1 31.0%), first division Jupiler Pro league (Q4 19.0% vs. Q1 6.9%), second division 1B (Q4 9.5% vs. Q1 8.6%) (Figure 3).

This is in line with the underdog hypothesis (11, 15). Since these players were selected for an elite team, the relatively young players had already “beaten the odds.” Secondly, these relatively young players trained and competed with (at a given point in time) better players which was beneficial for their soccer education (15). Gibbs et al. also found that the average career duration was longer for relatively young players (11). Moreover, Ashworth and Heyndels found that players born late after the cut-off date earn systematically more (15).

These superior psychological constructs and successful long-term development of relative young players can be related and may be explained by the “underdog hypothesis”, whereby being a relatively young player essentially facilitates long-term development by necessitating the player overcome the odds of the deep-rooted phenomenon of RAE, probably through being challenged by their older and more advanced peers (11, 30, 37). In the data collection, the achievement of senior professional status was noted; defined as signing a full-time professional contract for a minimum of one year. It is essential to better understand and gain more insights into why certain players might be more likely to be selected into an academy and to get more access to interesting competitions and training, and also why others might be more likely to successfully graduate and earn contracts as professional players. The current study showed not only further evidence of the RAE within a youth elite national soccer association but also provided evidence of the underdog hypothesis in national elite youth teams. This hypothesis only applied when relatively young athletes were selected for an elite team. It has elsewhere been detected in elite hockey players and national-level rugby and cricket players (11). Relatively young players are more likely to get higher value senior professional contracts, subsequently suggesting this may be due to the relatively young players developing superior psychological skills and technical expertise to compensate for their early physical disadvantage. It has been suggested that since they are selected to be elite players “against the odds”, they are more talented through being challenged by their older and more advanced peers (11, 35, 37, 58, 60). Relatively young players that are selected to play on an elite team also have the advantage of receiving higher quality soccer education.

This underdog hypothesis may suggest a reversal of the distribution bias in the youth to senior transition as an elite soccer player. This is indicative of the potential advantage to those chronologically younger players within an elite youth soccer academy (11). Specifically, the underdog hypothesis suggests that to be competitive and be retained in elite youth soccer academies, relatively young and late maturing players must either be creative and possess and develop superior technical, tactical, and psychological skills. This comparatively greater challenge, which is experienced by relatively young and later maturing players, is thought to facilitate and encourage the development of these superior skills (11). While superior psychological and technical/tactical skills might be masked through childhood and adolescence, they become more obvious in late adolescence and early adulthood when age and/or physical maturity are attenuated or reversed (66). Late maturing players even benefit from spending a longer period in childhood and adolescence and so on in different developmental stages that are each optimized for specific learning and motor skill development to become more creative as a soccer player (67). However, the underdog hypothesis will only be realized during development in youth soccer academies if relatively young and later maturing youth are selected into or retained within the soccer development youth process, being exposed to the RAE (11, 37). The importance of being exposed to challenges and the need to possess adaptive psychological and behavioral skills have been long established as essential requisites for developing excellence in senior soccer levels (22, 68, 69). Young relative age may still cause an underdog advantage in attributes including motivation, decision making, resiliency, and/or technical and tactical ability.

Consequently, by eliminating the RAE in youth academy soccer academies, the potential “underdog” benefits for later birth quartiles, through consistently engaging with their older peers, may also be removed. Through competing against relatively old and often more mature players within their annual chronological age group, relatively young players have to develop certain technical proficiencies and/or tactical awareness to be able to counteract this physical bias against relatively old players (11, 22, 23, 30, 70, 71). So, more concrete, a physically larger and stronger player may be able to easily dispossess a physically smaller, weaker opponent as a result of their physical dominance, thus a smaller, weaker late-mature player must create a technical or tactical solution to reduce this advantage and to compete and develop successfully. These younger, smaller, late-mature players must overcome “a system that discriminates against them”, by being more creative, skilled, and talented than their relatively larger counterparts to counteract their physical stature advantage (30, 70).

Furthermore, smaller and late-mature players which are often physically inferior throughout their youth development as a result of their younger age may have developed certain psychological constructs and skills, once primarily selected for youth academies towards adulthood, that allowed them to compete against their earlier mature opponents (2, 35). The underdog hypothesis demonstrated that the initial physical disadvantage may eventually contribute to the later psychological superiority when early differences in physical characteristics plateau towards the senior elite playing level (11, 37, 70). This is potentially through learning to “work and compete harder” and to be more creative, resulting in peer effects that facilitate resilience and improved motivation and self-confidence (22, 37). Thus, these psychological benefits and strong personality constructs like self-confidence, likely equip the chronologically relatively young players, or “underdogs”, to overcome subsequent obstacles and opponents and succeed at a senior professional level (70, 71). Further support for the underdog hypothesis can be provided by the fact that selected relatively young players benefitted from competitive play with older peers by getting more match- and training- related experience, augmenting their psychological advantage to compete successfully, compared to their earlier maturing equivalents.

The results also demonstrated that more players who were born in the first birthdate quarter, and examined eight years later, had not been retained by a club or had ended their career early (Q1 19.0% vs. Q4 14.3%) (Figure 3). The observation that more players of the first quarter ended early in their career may be associated with relatively lower scores on personality constructs such as motivation, winners’ mentality, and self-confidence (Tables 1, 2).

Comparing the top 20 players, who scored best on personality constructs, with the bottom 20 players, who scored worst on personality constructs, almost double the number the players in the top 20 (with strong personality constructs) received professional contracts as senior players (Top 20: 65% vs. bottom 20: 35%) (Figure 4). Even more players of the bottom 20 players (30%) ended their careers early compared with the top 20 players (0%), who scored best on personality constructs (Figure 4). So, an association between lack of superior personality constructs like motivation, self-confidence, and winners’ mentality and lack of challenge during their development may determine early “drop-out” as a soccer player. We do not expect that more players with higher scores for personality constructs are still involved in professional soccer (39, 56).

## Limitations

One of the strengths of this study is that the study population is quite large. Furthermore, it is one of the first studies to examine the potential relationship between birthdate quartile and personality constructs in elite soccer. Different components (RAE, 5 personality construct s, and long-term career development) were investigated, incorporating new and innovative strategies to eliminate the RAE within talent identification and development processes in academy football to retain all potentially talented players during their development. Although this study demonstrates early promise, there are still a few limitations in this study.

As noted above, the study population is a sample of the best Belgian youth elite players, which can explain the limited differences between the quartiles (Tables 1, 2). In addition, since the selection of players in the elite national youth team already happened retrospectively (before the data collection) this study can't test prospectively if the small differences in personality traits are one of the reasons for the RAE in this population. Also, the data originally were collected by the Royal Belgian Football Association for practice improvement, rather than for scientific research. In the psychologists’ observations of the players during matches, a lot of factors were therefore not standardized, such as the strength of the opponent, the final score, and the number of minutes of play for a specific player.

Another limitation to be stated is that the Mann–Whitney *U* test can only compare two groups, which makes it difficult to see a trend over the four quartiles. Also, medians were compared instead of means. As a result, few differences were perceived between groups. Nevertheless, medians were most appropriate since linearity of the data could not be assumed.

Finally, the use of the questionnaires, should be discussed. According to guidelines stating that psychological constructs should be evaluated by combining players’ self-rating and external expert rating in talent development and psychological analysis, the players performed a self-assessment by filling out the “Mental Potential Questionnaire” and the “Task & Ego Orientation in Sports Questionnaire” (TEOSQ) (40) in combination with observation during match play and an interview by external sports psychologists (27, 56). This self-assessment by the “Mental Potential Questionnaire” is nowadays replaced by valid questionnaires like the “Athletic Coping Skills Inventory” (43) and the “Big Five” (42). The Athletic Coping Skills Inventory (“ACSI”) is a highly validated psychology assessment that measures an athlete's psychological coping skills in seven key areas coping with Adversity, coachability, concentration, confidence and achievement motivation, goal setting and mental preparation, peaking under pressure and freedom from worry (43).

The five-factor model of the “Big Five” is a dimensional representation of personality structure that has gained widespread acceptance among personality psychologists to analyze personality disorder scales. It validates five factors neuroticism, extraversion, openness, agreeableness, and conscientiousness. It helps to guide athletes who are interested in gaining more understanding and clarity around these mental skills that can impact performance. Athletes will answer a series of questions surrounding different performance psychology components (42).

We highly recommend the inclusion of valid sports psychologists’ external ratings in addition to standardized self-report questionnaires (27). Within this perspective, there is a great potential in considering the psychological factors of players as predictors of current and future soccer performance and talent development and therefore clear recommendations and standards are needed to ensure an efficient and valid diagnostically assessment for clubs and associations within the youth talent development process (1, 27, 58). Nevertheless, beyond performance enhancement, there is much more to ethical, holistic talent development (60, 72, 73). We agree that in talent development, psychological constructs may also be important for promoting holistic athlete development to include players’ well-being, the formation of positive relationships, and non-sporting enhancements to develop as strong individuals and athletes (73). In the context of the assessment of personality constructs for talent development, we recommend combining different sources of information like the players’ self-assessment and the sport psychologists’ external ratings (27, 74). Integrating self- and external ratings would be essential in developing a “multitrait-multimethod” approach (27, 75). A “multitrait-multimethod” approach for psychological constructs in talent identification and development involves the validation of different psychological characteristics by applying a variety of methods. For example, for measuring and validating “motivation” of a player, a standardized questionnaire could be used, and coaches and other teammates can be asked to fill out standardized evaluation sheets with behavioral evaluations and interviews, behavioral observations by video analyses, or small-sided games by external experts can be added (72).

## Implications

This study shows that in the long run, relatively young soccer players who get selected for elite teams can turn their disadvantage into an advantage. The problem is that due to the RAE only a few relatively young players get the opportunity to show their worth (6, 9). The results of this study demonstrate that relatively young players also have a lot of potential to become successful soccer players. For this reason, scouts, coaches and the players themselves must acknowledge their strengths. By putting more emphasis on their strengths than on their weaknesses, relatively young players may be less discouraged and less likely to drop out before they reach a certain level of success (1, 27, 59). By frequent selection for national teams and successful clubs, youth players are exposed to better coaching and face better opponents; are implemented in higher and more prestigious competition levels, which would increase self-confidence and motivation. Many promising youth players have been overlooked by deselection in their early childhood because they suffered from a relative age and maturity disadvantage, which in the long-term will lower the overall multifactorial quality of professional sports teams, while youth players with proper technical and tactical attributes are being overlooked at an early age due to a lack of physical development and maturity that is only related to the period of the selection year in which they were born (6, 12, 19). The realization of possible solutions to the relative age (and maturity selection bias) is essential for efficient youth talent development processes. The awareness of staff and institutions is frequently inadequate to reduce this selection bias (44, 61). In their interactions with relatively young players, coaches should always concentrate on the long-term player's improvement in multifactorial constructs, rather than conducting unfair comparisons with the relatively old peers and focusing on short-term aims and isolated outcome factors like winning.

Different solutions to tackle relative age- and maturity-related bias have been proposed throughout the years, including changing and rotating the cut-off dates (44, 61), installing sport-specific cut-off dates, age-ordered numbered soccer shirts (76), birthday banding, the bio-banding method, whereby players are grouped according to their maturation status (3, 10, 77–82). Despite the relative success of some of these methods to reduce RAE (i.e., birthday and bio-banding) and maturity selection bias (i.e., bio-banding), some proposed remedies are difficult to implement in the daily structure of training programs and game formats. The recently proposed reallocation method showed to remove evidence of the relative age effect of players when compared to their distribution before reallocation and reduced the between-player variation in maturity, anthropometric and physical characteristics when compared to chronologically categorized playing ages (16, 18). It may be hypothesized that by eliminating Matthew effects and Pygmalion effects, RAEs could also be minimized, or at least influenced, but further research is necessary. It could be interesting for future research to investigate if RAEs could be reduced by using the reallocation method in our study population.

Awareness about the existence and possible consequences of the RAE in soccer settings is essential during the identification and development of youth soccer players and should be analyzed carefully in all their facets to minimize the loss of potential talent in the long-term and for the best development of all players on equal terms (9, 19, 44). It is unclear whether the avoidance of early or frequent deselection as a youth player within a talent pathway is beneficial for achieving long-term expertise, because of the underdog hypothesis (11, 37, 70). The long-term purpose of a youth soccer academy should be to identify and then develop talented youth soccer players towards strong multifactorial future performance abilities, in which attention should rather concentrate on the long-term course of multifactorial development, rather than focusing on current isolated performance abilities like physical characteristics (1, 2, 27, 35). In this study, a reversal of the distribution bias in the youth to senior transition in the context of development to a professional player was observed, indicative of the potential advantage and abilities of those chronologically relatively young players. An interesting issue of this current study is that by eliminating the RAE in academy youth soccer the potential benefits created by the underdog hypothesis for relatively young players, through consistently engaging with their older peers, may also be removed. By playing against relatively old, more mature players within their chronological age group, relatively young or late mature players have to develop certain technical skills and tactical awareness to be able to counteract this bias based on lower physical characteristics (5, 11, 30, 70, 71). The relatively old and more mature player with stronger physical characteristics may be able to easily counteract a relatively young player, which is smaller and less strong as a result of their physical dominance, thus relatively young or late mature players must create a technical or tactical solution to reduce this advantage. Relatively young players must overcome a system that discriminates against them, by being more talented and creative than their relatively large counterparts to counteract their physical stature and body mass advantage. Therefore, this underdog hypothesis, where the initial physical disadvantage may eventually contribute to the later multifactorial superiority when early differences in size equalize towards development as a professional senior player (11, 30, 37, 70). This is potentially through learning to compete in difficult “unequal” situations, resulting in peer effects that facilitate self-confidence, resilience, and improved motivation. Thus, these psychological values and benefits likely advantage the chronologically younger players, or “underdogs”, to overcome subsequent obstacles during their development and succeed at the senior professional level by receiving professional contracts (71). Therefore, the main issue would be how youth soccer academies get the “best of both worlds” concerning moderating the RAE whilst also gaining the benefits and values of the underdog hypothesis. Current strategies to create an occasional underdog hypothesis in youth player academy development appear still unexplored (11, 37). This underdog effect could be occasionally created in training formats of youth academy settings by “playing-up” a chronological age group to facilitate more efficient and multifactorial development of relatively old players by creating an “underdog effect” in an older age group. Furthermore, these inventions may also reduce the widely reported high drop-out rates amongst late mature players (19) by providing more selection opportunities for relatively young and late mature players into an elite youth soccer academy at an early age. Even by “playing-down” a chronological age group, it may also offer a more suitable developmental setting for late mature players whilst they compete with their chronologically older peers, whilst also providing a more challenging environment for early birthdate quartiles in a younger age group. Therefore, occasionally implementing in training formats of youth soccer academies a “flexible chronological approach” to players, offers relatively old and relatively young players the opportunity to play up and play down an annual age group respectively, as opposed to fixed chronological bandings. This transient rotating system of “flexible approach” may also be applied in the recently presented maturity-related system of reallocated groups to create an “underdog effect”.

The Royal Belgian Football Association is working with “future youth teams”, that are made up from late mature players. In this way, late maturing talented Belgian players have the opportunity to benefit from the high-quality training and competition in order to combat the problems of maturity and growth. Occasionally these teams are implemented in training and competitions formats with older players as “flexible chronological approach”.

Because of the RAE, higher drop-out rates are observed among relatively young and late-mature players (6, 9, 19). However, the results of this study show that relatively young players have more opportunities to reach a status as professional senior players in the long term.

In the context of the RAE, it is essential to acknowledge the limited opportunities for relatively young and late mature players to be selected in an elite youth soccer academy, thus emphasizing the dual responsibility and importance of soccer academy staff and scouts to be aware of the RAE in youth soccer academies in order to prevent loss of talent by early drop-out and to develop youth players holistically/ multiskilled to a professional talented senior soccer player.

The least mature players and relatively young players within an age group may have a greater need to possess superior technical/tactical or psycho-behavioral skills than those relatively old counterparts. Because it is essential to highlight the potential benefits of later maturation in soccer, it's equally important to consider any possible disadvantages associated with early maturity. Due to the pressure to succeed and avoid being released, early maturing players may be encouraged only to rely on their stronger physical and functional advantages at the expense of their psychological and technical/tactical development (3, 4, 7). In the context of the transient nature of physical and functional advantages, early maturing players will also be unable to rely upon these physical attributes at the adult level during their development as youth soccer elite players. Therefore, youth soccer academies need to create multifactorial learning environments that encourage early maturing players to develop more adaptive creative skill sets (technical, tactical, psychological) and not only rely on their physicality. Recently proposed strategies such as the reallocation method (16, 18) future teams and bio-banding (10, 77), in which players are periodically grouped by maturity status rather than chronological age, will expose early maturing players to a greater challenge and to provide the same learning conditions and creativity that late maturing players experience regularly in chronologically based groups. In case early mature players compete in reallocated or bio-banded formats, they are less able to rely on their physical and functional qualities and are forced to use their technical, tactical, and psychological skills (3, 10, 16, 18, 83).

Sports psychologists can use their expertise to support early maturing players in such contexts by improving their psychological provision by evaluating their psychological assessment, teaching early maturing players how to create self-confidence and more adaptive self-regulatory skills to optimize their physical, psychological, technical, and tactical development (4, 27, 56). By limiting maturity-associated variation in physical characteristics like stature and body mass, bio-banding and reallocation may give late maturing players greater opportunity to use and demonstrate their technical, physical, and psychological attributes (3, 10, 16, 18, 83). Moreover, maturity-related grouping encourages a less physical and more technical and tactical style of play and will develop more multifactorial skilled and talented youth elite players in soccer academies. Maturity-related grouping strategies will reveal more technical-skilled soccer with more passing and dribbling in the maturity-matched format and will create youth soccer players with stronger personality constructs, that are essential to reach professional status in the long term (10, 37, 83). Academy soccer staff like coaches and scouts need to be aware of the RAE which is implemented in youth soccer academies by selection procedures and need to give more attention and relevance to psychological strength when evaluating player performance without being biased by maturity-associated characteristics (4, 27, 35). The RBFA puts a lot of effort into education and awareness about the RAE by coaches and scouts.

The Union of European Football Associations (UEFA) Financial Fair Play regulation states that all European professional soccer clubs are to operate and manage youth soccer development within their financial means (51). Because of the importance to develop “home-grown” youth talent to compete at professional senior level soccer and reduce club financial outgoings on imported talented players, there are a need for domestic professional soccer clubs to install ongoing talent (de)selection strategies that are free from (sub)conscious, transient, maturity related selection bias to prevent early drop-out of talented late mature players who have more opportunities to develop as a professional senior player. Another strategy in case of interesting transfers may be to focus as youth soccer academy on talented late mature or relatively young players with initial lower financial value on the transfer market because of inferior physical characteristics, but who have more opportunities to develop as a professional senior player on the long-term because of their stronger technical, tactical and psychological skills. Therefore, good knowledge, guidelines, and awareness of the RAE and holistic characteristics of potential talented relatively young youth soccer players is essential for recruitment practitioners to reduce over-selection of early-maturing players and recruit “talented”, multi-skilled relatively young players to create a bigger talent pool for elite youth soccer academies (1, 2, 4, 27, 35, 44, 58).

To successfully implement the diagnostic psychological assessments and interventions for potential talented youth players, we believe that additional support from experts in the field of sports psychology should be provided to clubs, elite youth soccer academies, and coaches. On the level of soccer formats in clubs and national associations, coach and scouting education programs could embed basic psychological diagnostic and interventional assessments within the standard curriculum to get some more skills around the development of personality characteristics in youth soccer players (1, 27, 32, 35, 58, 62, 73). To provide evidence-based insights into the correct and efficient psychological diagnostic principles, quality criteria, and potential interventions, in line with licensing requirements, cooperation with sports psychologists for clubs and soccer associations would be valuable. A lot of elite youth soccer academies consult sports psychologists to support the players and coaches in implementing the psychological assessments and goals set for their players’ personality and well-being as well as efficient psychological skill development, especially for early mature players (27). Sports psychologists should not only offer psychological diagnostic assessments and interventions in talented youth development programs but also support staff and players to improve daily communication and functional performance in match and training formats. Licensed sports psychologists can help in relating relevant personality characteristics to theory-based psychological constructs and ensure an efficient diagnostical assessment of youth players. Sports psychologists belong to the staff of national youth soccer teams in Belgium, in order to support the psychological skill development of all elite youth players and improve functional performances in both match and training formats. Secondly, sports psychologists can support coaches’ assessments in daily communication with players in training formats by providing feedback on the psychological intervention process of player development and evaluating results (27, 39, 57). Concerning the long-term development of youth academy players, applying a multitrait-multimethod approach and repeated measurements by the combination of questionnaires, interviews, and observations can provide the basis for an integrated, individual, long-term development plan for the players (27, 39). Through evidence-based diagnostic assessments and theory-based specific interventions for early- and late-mature players, sports psychologists should help develop the players’ psychological skills according to their individual needs based on the RAE and diagnostic assessments (84). By awareness about the importance of personality characteristics in the development of youth elite soccer players, educating and supporting coaches in psychological diagnostic assessments, and intensive cooperation with sports psychologists, higher quality standards concerning psychological diagnostics and interventions in youth talent development programs could be reached. Consequently, improved and evidence-based diagnostic assessments and personal intervention programs for the psychological characteristics of talented players can, in turn, result in the better long-term performance of elite youth soccer players, which is beneficial for all stakeholders in talent development formats (1, 27, 35, 58, 60, 73, 84).

## Future directions for research

Concerning future research, it may be interesting to perform a longitudinal prospective study with a more extensive cohort of youth soccer players of different age categories and from different playing levels, before they are selected for any elite soccer team. This study format would explore in greater depth the association between personality characteristics and RAE because of Pygmalion, Galatea, and Matthew effects by the inclusion of psychological assessment of late mature or relatively young youth soccer players who eventually would not be selected in elite youth soccer academies and would drop out on the long-term.

Moreover, it would also be interesting to observe whether personality constructs change throughout an elite youth player's career due to his experience and holistic development as a player.

Further, future research can be performed to get some more insight into differences and progression in personality constructs between youth soccer players in more maturity-related grouping strategies like the reallocation method or bio-banding. It would also be interesting to observe if coaches nowadays take care of psychological constructs and mental well-being of their players.

Finally, it would be intriguing to observe if specific personal intervention programs for psychological constructs of talented elite youth soccer players, based on their psychological diagnostic assessment, would improve their opportunities to reach a senior professional status in the long term.

## Conclusion

Academy soccer players’ development and talent identification settings are multifaceted and complex. Both require soccer practitioners to make informed judgements and evaluations relating to players’ physical, technical, tactical, and psychological characteristics, which have been associated with greater development and performance outcomes as elite youth players and in their successful transition from youth academy level to senior professional status. Relative age and maturity-related selection bias have been offered as causal factors for the overrepresentation of players who are either relatively old and/or early maturing. This study demonstrated a clear RAE in a group of 151 male elite Belgian youth soccer players. Early maturing players are often characterized as possessing temporary, maturity-related enhancements in anthropometric and physical fitness characteristics, consequently, they are often perceived as “more talented” by soccer practitioners. Relatively young players must overcome this selection bias, by being more talented and creative than their relatively large counterparts to counteract their physical advantage. Results of the study showed that once they reach adulthood, because of being creative and diverse with good technical and tactical skills, they also may have developed stronger psychological constructs like self-confidence and a winners’ mentality that previously allowed them to compete. Relatively old players scored better on personality constructs like team orientation. The association between personality characteristics and RAE can also be explained by the Pygmalion, Galatea, and Matthew effects. The underdog hypothesis, where the initial physical disadvantage may eventually contribute to the later multifactorial superiority, provides relatively young players with more opportunities for successful development towards a professional senior player status, as they more frequently sign professionally as a relatively young player. Therefore, strategies to counteract the RAE are essential, to prevent early drop-out of late maturers and often more talented players with more opportunities to reach professional status.

However, underdog effects cannot be ignored whilst considering the socio-environmental dynamics when incorporating new and innovative strategies to eliminate the RAE within talent identification and development processes in academy soccer. Thus, it is suggested that soccer recruitment practitioners should act with caution when creating strategies to eliminate the RAE by eliminating the Galatea and Matthew effects, as doing so may also eradicate the underdog hypothesis. Therefore, occasionally implementing a “flexible chronological approach” in training formats of youth soccer academies by offering relatively old and relatively young players the opportunity to play up and play down a chronological or maturity-based age group respectively, as opposed to fixed chronological bandings, may help to counteract the underdog hypothesis.

Creating more awareness about the importance of personality constructs in the future development of youth elite soccer players, educating and supporting coaches in psychological diagnostic assessments and intensive cooperation with sports psychologists, may result in higher quality standards concerning psychological diagnostics and more specific interventions in youth talent development programs.

However, further research into the proposed solutions for the RAE, like maturity-related grouping as the reallocation method, and influences on the development of personality constructs, is required, to ensure there is a continued emphasis on creating the right environment for every player to develop to their full potential in elite youth soccer academies, taking into account side effects like the underdog hypothesis.

## Data Availability

The raw data supporting the conclusions of this article will be made available by the authors, without undue reservation.
